# Endoplasmic reticulum stress‐induced exosomal miR‐27a‐3p promotes immune escape in breast cancer via regulating PD‐L1 expression in macrophages

**DOI:** 10.1111/jcmm.15367

**Published:** 2020-07-16

**Authors:** Xiaoli Yao, Yi Tu, Yulin Xu, Yueyue Guo, Feng Yao, Xinghua Zhang

**Affiliations:** ^1^ Department of Breast and Thyroid Surgery Renmin Hospital of Wuhan University Wuhan China; ^2^ Department of Thoracic Surgery Renmin Hospital of Wuhan University Wuhan China

**Keywords:** breast cancer, endoplasmic reticulum, exosomes, macrophages, MicroRNA‐27a‐3p, programmed cell death‐Ligand 1, tumour immune evasion of breast cancer cells

## Abstract

Immune escape of breast cancer cells contributes to breast cancer pathogenesis. Tumour microenvironment stresses that disrupt protein homeostasis can produce endoplasmic reticulum (ER) stress. The miRNA‐mediated translational repression of mRNAs has been extensively studied in regulating immune escape and ER stress in human cancers. In this study, we identified a novel microRNA (miR)‐27a‐3p and investigated its mechanistic role in promoting immune evasion. The binding affinity between miR‐27a‐3p and MAGI2 was predicted using bioinformatic analysis and verified by dual‐luciferase reporter assay. Ectopic expression and inhibition of miR‐27a‐3p in breast cancer cells were achieved by transduction with mimics and inhibitors. Besides, artificial modulation of MAGI2 and PTEN was done to explore their function in ER stress and immune escape of cancer cells. Of note, exosomes were derived from cancer cells and co‐cultured with macrophages for mechanistic studies. The experimental data suggested that ER stress biomarkers including GRP78, PERK, ATF6, IRE1α and PD‐L1 were overexpressed in breast cancer tissues relative to paracancerous tissues. Endoplasmic reticulum stress promoted exosome secretion and elevated exosomal miR‐27a‐3p expression. Elevation of miR‐27a‐3p and PD‐L1 levels in macrophages was observed in response to exosomes‐overexpressing miR‐27a‐3p in vivo and in vitro. miR‐27a‐3p could target and negatively regulate MAGI2, while MAGI2 down‐regulated PD‐L1 by up‐regulating PTEN to inactivate PI3K/AKT signalling pathway. Less CD4^+^, CD8^+^ T cells and IL‐2, and T cells apoptosis were observed in response to co‐culture of macrophages and CD3^+^ T cells. Conjointly, exosomal miR‐27a‐3p promotes immune evasion by up‐regulating PD‐L1 via MAGI2/PTEN/PI3K axis in breast cancer.

## 1 INTRODUCTION

Breast cancer is the most prevalent malignancy among females and its incidence and mortality rate are predicted to increase in the coming years.^1^ Advances in breast cancer screening have been updated internationally and the development in genomics helps to establish a new molecular categorization of breast cancer.^2^ However, conservation surgery accompanied by radiotherapy was likely to relapse and it's largely dependent on patient age and other clinicopathological factors.^3^ Recently, immunotherapy has emerged as a promising option for treating breast cancer, wherein a patient's own immune system is activated to combat breast cancer.^4^

microRNAs (miRs) are important post‐transcriptional regulators of gene expression and involved in cellular gene regulatory pathways.^5^ In addition, miRNA‐containing exosomes released from cancer cells are known to contribute to tumour growth and progression.^6^ miR‐27a‐3p has been identified as an oncogenic RNA in multiple cancers including gastric and colorectal cancer, and is one of the highly‐enriched miRNAs found in exosomes of breast cancer cells.^7^, ^8^, ^9^ Bioinformatics analysis has predicted the possible binding sites between miR‐27a‐3p and the mRNA of membrane‐associated guanylate kinase inverted 2 (MAGI2), a scaffold protein required for phosphatase and tensin homolog (PTEN) stability. MAGI2 has been previously implicated in regulating the migration and evasion of the breast cancer cells.^10^, ^11^ In particular, MAGI2 has been found to regulate PTEN stability.^12^ The PTEN‐PI3K/AKT axis plays an essential role in cell proliferation, migration and apoptosis in multiple myeloma as well as many other cancers.^13^ In addition, programmed cell death‐Ligand 1 (PD‐L1), a protein exhibiting immune‐inhibitory effect, is commonly found expressed on cancer cells and mediates immune escape.^14^, ^15^ Therefore, we examined if breast cancer cell‐derived exosomal miR‐27a‐3p is involved in immune evasion by regulating PD‐L1 expression.

## 2 MATERIALS AND METHODS

### 2.1 Ethics statement

The study was carried out under the approval of the Ethics Committee of Renmin Hospital of Wuhan University and all procedures followed the tenets of the Declaration of Helsinki. All patients signed written informed consent prior to participation in the study.

### 2.2 Bioinformatics

TargetScan (http://www.targetscan.org/vert_71/) was used to predict the correlation of and possible binding sites between miR‐27a‐3p and MAGI2.

### 2.3 Sample collection and cell culture

Tumour tissues of primary breast cancer and paracancerous tissues (≥5 cm from the tumour margin) were collected from 26 triple‐negative breast cancer patients (mean age of 51.23 ± 3.18 years) who underwent tumour excision between 2013 and 2017 at the Renmin Hospital of Wuhan University. All enrolled patients were diagnosed with metastatic or locally advanced triple‐negative breast cancer. Validation of oestrogen receptor (ER) and epidermal growth factor receptor 2 (HER2) negativity (ER < 10% and HER2 0, 1 or 2 in the absence of amplification assessed by in situ hybridization) was performed with a biopsy of a metastatic lesion or recurrence in the breast.^16^ Breast cancer tissues and paracancerous tissues were fixed using formaldehyde and embedded in paraffin for diagnosis. The histopathology of breast cancer sections was evaluated using Edmondson‐Steiner grading. Patients were classified into stage I (10), II (7) and III (9) based on the World Health Organization and tumour‐node‐metastasis staging system. MCF‐7, BT474, MDA‐MB‐23, MCF‐10A and THP‐1 breast cancer cell lines were obtained from American Type Culture Collection (Manssas, VA, USA). Cells were cultured in the Dulbecco's modified Eagle's medium (DMEM) containing 10% foetal bovine serum (FBS; Gibco, Carlsbad, CA), followed by culture in 5% CO_2_ at 37°C with relative humidity of 95%. Cells were passaged at confluency of approximately 90%.

### 2.4 ER stress measurement

Endoplasmic reticulum stress‐related proteins including GRP78 (ER master stress regulator) and IRE1α, PERK, ATF6 (ER stress sensors) were measured to quantify the degree of ER stress. The percentage of positive cells was graded (criteria: 0 indicates negative; 1 indicates ≤ 10%; 2 indicates 11%‐50%; 3 indicates 51%‐75%; 4 indicates > 75%). Staining intensity was measured in 5 random fields (0 stands for no staining; 1 for light yellow; 2 for pale brown; 3 for dark brown). Endoplasmic reticulum stress‐related protein expression was determined as the percentage of positive cells multiplied by staining intensity. A score of 5 was used as the threshold to define low or high expression level for GRP78, while a score of 3 was used for IRE1α, PERK and ATF6.

### 2.5 Cells treatment

Breast cancer cells (4 × 10^5^ cells/well) were seeded in 6‐well culture plates and transfected using lipofectamine 2000 (11668‐019, Invitrogen, New York, California, USA) according to the manufacturer's instructions. When cell confluence reached 80%‐90%, except for cells for control (without transfection), cells were transfected with plasmids containing miR‐27a‐3p mimic, miR‐27a‐3p inhibitor, overexpression (OE)‐MAGI2, OE‐PTEN, small interfering RNA (si)‐MAGI2, si‐PTEN, OE‐MAGI2 + si‐PTEN, or their relative negative control (NC). Transfection sequences and plasmids were purchased from Shanghai GenePharma Co., Ltd. (Shanghai, China).

### 2.6 RNA isolation and quantitation

Total RNA was extracted using RNeasy Mini Kit (Qiagen, Valencia, CA, USA) and RNA was reverse‐transcribed using First Strand cDNA Synthesis Kit (RR047A, Takara, Japan). SYBR Premix EX Taq Kit (RR420A, Takara) was used for the reactions involving mRNA, while miScript SYBR Green PCR Kit (218073, Qiagen, Hilden, Germany) was used for miRNA. Real‐time quantitative PCR was performed using ABI7500 Real‐Time PCR System (7500, ABI, Foster City, CA, USA). All primers (Table 1) were synthesized by Sangon Biotech (Shanghai, China). Relative expression was calculated using the 2^−ΔΔCt^ method. Glyceraldehyde‐3‐phosphate dehydrogenase (GAPDH) was used as internal reference for mRNA, while U6 was used as reference for miRNA.

**TABLE 1:** Primer sequences for RT‐qPCR

Targetgene	Primersequences(5′‐3′)
GRP78	F: 5′‐TAGCGTATGGTGCTGCTGTC‐3′
	R: 5′‐TTTGTCAGGGGTCTTTCACC‐3′
PERK	F: 5′‐AGGACAGAGGGGACAGAGTTG‐3′
	R: 5′‐TAATGACCTTTTCTTCCCTGCTCC‐3′
IRE1α	F: 5′‐TAGTCAGTTCTGCGTCCGCT‐3′
	R: 5′‐TTCCAAAAATCCCGAGGCCG‐3′
ATF‐6	F: 5′‐CTGATTCTCATTCAGGCTTCTCAC‐3′
	R: 5′‐GAAGGCATCCTCCTTGCTGTT‐3′
miR‐27a‐3p	F: 5′‐ATGGTTCGTGGGTTCACA‐3′
	R: 5′‐GTGGCTAAGTTCCGACG‐3′
MAGI2	F: 5′‐TCCGGCTCAAGTGTGTCAAG‐3′
	R: 5′‐AGGTTGTCACGAATGATTTGCT‐3′
PTEN	F: 5′‐CACAGAATTCCAGACATGACAGCCATCATC‐3′
	R: 5′‐GTCGATCCTCATGGTGTTTTATCCCTCTTG‐3′
PI3KR1	F: 5′‐CAGCAACCTGGCAGAATTACGA‐3′
	R: 5′‐TGACAGGATTTGGTAAGTCCAGGAG‐3′
GAPDH	F: 5′‐CGACTTCAACAGCAACTCCCACTCTTCC‐3′
	R: 5′‐TGGGTGGTCCAGGGTTTCTTACTCCTT‐3′
U6	F: 5′‐ATACAGAGAAGATTAGCATGGCCCCTG‐3′
	R: 5′‐ACACGCAAATTCGTGAAGCGTTCCATATTT‐3′

Abbreviations: F, forward; R, reverse.

### 2.7 Western blot assay

The cells were collected and lysed using radioimmunoprecipitation assay (RIPA) lysis buffer containing phenylmethylsulphonyl fluoride (PMSF). Bicinchoninic acid (BCA) protein assay kit (23227, ThermoFisher Scientific, New York, USA) was used to quantify the protein concentration. Proteins were separated by SDS‐polyacrylamide gel electrophoresis and then transferred onto a polyvinylidene fluoride membrane (Millipore, Milford, MA, USA). The membrane was blocked with 5% bovine serum albumin (BSA; 1 hour) and incubated overnight with primary antibody against: GRP78 (1:500, ab32628), PERK (1:1000, ab65142), IRE1α (1:500, ab37073), ATF‐6 (1:200, ab23760), PDL‐1 (1:500, ab237726), MAGI2 (1:500, ab97343), PTEN (1:500, ab31392), PI3K (1:1000, ab191606), AKT (1:500, ab179463), p‐AKT (1:500, ab131443) and GAPDH (1:5000, ab8245). On the following day, the membrane was incubated with goat anti‐rabbit IgG labelled with horseradish peroxidase (1:20 000, ab205718) for 1.5 hours. All antibodies were obtained from Abcam (Cambridge, UK). The developer was then added (NCI4106, Pierce, Rockford, IL, USA) for visualization. ImageJ 1.48u software (Bio‐Rad, Hercules, CA, USA) was used to analyse relative protein expression by calculating the ratio of the intensity of target bands relative to GAPDH.

### 2.8 Immunohistochemistry

Paraffin‐embedded sections were dewaxed using xylene, hydrated using gradient ethanol and incubated in PBS containing 0.5% Triton at room temperature for 20 minutes. The antigen was retrieved under low pressure for 2 minutes, boiled in 0.01 mol/L citrate buffer containing pH6.0 at 95°C for 20 minutes, and immersed in 3% H_2_O_2_ for 15 minutes to block endogenous peroxidase. Sections were sealed by sealing fluid with 3% BSA. The sections were added with diluted primary antibody against: GRP78 (1:200, ab21685), PERK (1:1000, ab65142), IRE1α (1:100, ab37073), ATF‐6 (1:50, ab37149), CD68 (1:50, ab125212), PD‐L1 (1:50, ab238697) and GAPDH (1:5000, ab8245), followed by incubation for 2 hours at 37°C. Biotin‐labelled goat anti‐rabbit IgG was added (1:500) and incubated for 20 minutes at 37°C, followed by counterstaining using haematoxylin (Shanghai Fusheng Industrial Co., Ltd., Shanghai, China) for 4 minutes, dehydration, clearing and mounting. All antibodies were obtained from Abcam (Cambridge, UK). The sections were then viewed under a microscope and scored in a double‐blind manner by two independent examiners.

### 2.9 Immunofluorescence

Breast cancer cells were fixed in 4% polyformaldehyde for 30 minutes and embedded in paraffin, followed by permeabilization using 0.5% Triton X‐100 (Sangon Biotech). Normal goat serum (Solarbio, Beijing, China) was added. Primary antibody against GRP78 (1:100, ab32628), PERK (1:1000, ab65142), IRE1α (1:100, ab37073), ATF‐6 (1:50, ab37149) and GAPDH (1:5000, ab8245), followed by culture at 4°C overnight. The following day, diluted secondary antibody, Alexa Fluor 647‐labelled donkey anti‐rabbit (ab150075; 1:400) was added for additional incubation at 37°C for 1 hour. These antibodies were obtained from Abcam. Then, 4′,6‐diamidino‐2‐phenylindole (Beijing Biodee Biotechnology Co., Ltd.) was added to the cells and incubated without exposure to light for 5 minutes. The cells were then blocked using anti‐fluorescence quenching blocking solution and a fluorescence microscope (TE2000, Nikon, Tokyo, Japan) was finally used for photography.

### 2.10 Flow cytometry

Cells were stained with anti‐CD68 (1 μg/10^6^ cell; AbD Serotech, Oxford, UK) conjugated with fluorescein isothiocyanate (FITC). Following incubation for 30 minutes at 4°C, cells were fixed with PBS containing 1% polyformaldehyde and cell apoptosis was measured using FACSCalibur apoptosis kit (Becton Dickinson, San Diego, CA, USA). Homotype antibodies were used to control non‐specific staining and WinMDI 2.8 software (J. Trotter, The Scripps Research Institute, La Jolla, CA) was employed to analyse at least 10 000 cells.

### 2.11 Cell apoptosis assay

Annexin V‐FITC/PI (Annexin V/PI) assay was used to determine cell apoptosis. The cell suspension (1 × 10^6^ cells/mL) was collected in a 10 mL centrifugal tube and centrifuged (503.1 *g*) twice for 5 minutes. Labelling solution (100 µL) was used to resuspend cells and the cells were incubated without exposure to light for 15 minutes. After a wash in PBS and centrifugation (503.1 *g*), the cell pellets were suspended in fluorescence FA‐FLOUS solution and incubated for 20 minutes at 4°C. FITC fluorescence was recorded using a FACScan (FACSCalibur, Becton Dickinson, USA) flow cytometer and analysed using Flowcytomix Pro software.

### 2.12 Enzyme‐linked immunosorbent assay (ELISA)

Tissues (50 mg) were immersed in 0.5 mL pre‐cooled PBS, followed by an ice bath and then homogenized using a high‐speed homogenizer for 2 minutes. The tissues were then centrifugated at 3000 r/min for 10 minutes at 4°C and the supernatant was collected. An ELISA kit (69‐50049; Mershack Biotechnology Co., Ltd., Wuhan, China) was used to measure vascular endothelial growth factor (VEGF) concentration. A microplate reader (Synergy 2, BioTek, Winooski, VT, USA) was used to assess the absorbance (A) at 450 nm. Standard concentration was set as x‐label, A value as y‐label. The regression equation of standard curve was calculated to determine the target protein concentration.

### 2.13 Dual‐luciferase reporter assay

The 3′‐untranslated region (3′UTR) of MAGI2 was synthesized. Wild type (WT) and mutant (MUT) of MAGI2 were prepared according to the seed sequence. MAGI2‐WT and MAGI2‐MUT were then inserted into the PmirGLO vector. PmirGLO‐MAGI2‐WTand PmirGLO‐MAGI2‐MUT were provided by GenePharma Co., Ltd. (Shanghai, China). mimic NC and miR‐27a‐3p mimic were co‐transfected with PmirGLO‐MAGI2‐WT and PmirGLO‐MAGI2‐MUT, respectively into HEK293T cells, followed by 48‐hour culture. The cells were then collected and a dual‐luciferase kit from Genecopoeia (D0010; Solarbio, Beijing, China) was employed to measure luciferase activity. GLomax20/20 Luminometer fluorescence detector from Promega (E5311, Shanxi Zhongmei Biotechnology Co., Ltd., China) was used to determine the fluorescence intensity.

### 2.14 Extraction and characterization of exosomes from breast cancer cells

Breast cancer cells MCF‐7 and MDA‐MB‐231 were transfected with miR‐27a‐3p mimic, miR‐27a‐3p inhibitor and with corresponding NCs after 24 hours. The cells were seeded and cultured overnight in serum‐free DMEM medium. When the cells reached 80%‐90% confluence, they were cultured in serum‐free DMEM for 24 hours and supernatant was collected. Cell debris was detached by centrifugation at 1257.8 *g* for 20 minutes at 4°C, followed by high‐speed centrifugation of the supernatant at 5031 *g* for 1 hour at 4°C. Serum‐free DMEM medium containing HEPES (PH = 7.4, 25 mmol/L) was used to suspend the precipitation. High‐speed centrifugation was repeated with supernatants discarded, and finally the precipitate was stored at −80°C.

Transmission electron microscope was applied to evaluate exosomes. Exosomes (30 μL) were added to a copper net and 30 μL phosphotungstic acid (pH = 6.8) was added, followed by counterstaining for 5 minutes, and finally photographed using a transmission electron microscope.

Flow cytometry was adopted to detect surface markers of exosomes CD63 content. Specifically, breast cancer cells‐transported exosomes were digested using pancreatin, centrifugated at 503.1 *g* for 5 minutes and were triturated into a single cell suspension. The cells were aliquoted and stored in 1.5 mL EP tube. PBS (1 mL) containing 1% BSA was used to triturate cells, followed by incubation for 30 minutes to block non‐specific antigens. PBS contained in 200 µL/EP tube was then used to resuspend cells and each group was added with CD63‐PE antibody (ab234251, PE, Abcam), followed by culture for 30 minutes. Samples without antibody were used as controls, and PE‐labelled anti‐human IgG was used as a homotype control. Thereafter, PBS containing 1% BSA was used to suspend cells and Guava easyCyte™ system flow cytometry was used to determine surface marker CD63 expression. Western blot was used to measure exosome surface markers CD63 (ab216130), CD9 (ab223052), CD81 (ab59477) and Tsg101 (ab30871) and negative marker GRP94 (ab13509). All antibodies were obtained from Abcam. miR‐27a‐3p expression in exosomes was determined using RT‐qPCR.

### 2.15 Co‐culture of breast cancer cells‐transported exosomes and macrophages

Breast cancer cells‐transported exosomes were labelled using PKH‐67 (green; MINI67‐1KT, Sigma‐Aldrich, St Louis, MO, USA), strictly following kit instructions and co‐incubated with macrophages (50%‐60% confluence) in 24‐well plates for 48 hours. Exosomes and macrophages were treated with plasmids containing Exo‐miR‐27a‐3p mimic, Exo‐miR‐27a‐3p inhibitor, and its corresponding NC, and observed using an inverted fluorescence microscope.

### 2.16 Breast cancer cells induced by tunicamycin

To induce ER stress, MDA‐MB‐231 and MCF‐7 breast cancer cells were seeded in 24‐well plates and cultured in eagle's minimum essential medium (EMEM) containing 10% FBS. Tunicamycin (1.0 μg/mL) was added into EMEM containing 2% FBS and cells were cultured for 7 days, with cell counting performed every 24 hours.

### 2.17 Macrophages stimulated by phorbol 12‐myristate 13‐acetate (PMA)

To differentiate macrophages, THP‐1 cells (1 × 10^6^ cells/well) were cultured in 6‐well plates and treated with 100 nmol/L PMA for 48 hours. Cell morphology was observed, and images of phase difference were collected using an inverted microscope (TE2000, Nikon, Tokyo, Japan).

### 2.18 Statistical analysis

All data were processed and analysed using SPSS 21.0 statistical software (IBM Corp., Armonk, New York, USA). Measurement data were expressed as mean ± standard deviation. Data conforming to normal distribution and homogeneity of variance in intergroup settings were compared using paired *t* test, while comparisons between two groups were conducted using unpaired *t* test and one‐way analysis of variance (ANOVA) was utilized to compare data among multiple groups, followed by Tukey's post hoc test. *P* < 0.05 was considered statistically significant.

## 3 RESULTS

### 3.1 ER stress‐induced macrophage infiltration and up‐related PD‐L1 expression

**FIGURE 1:**
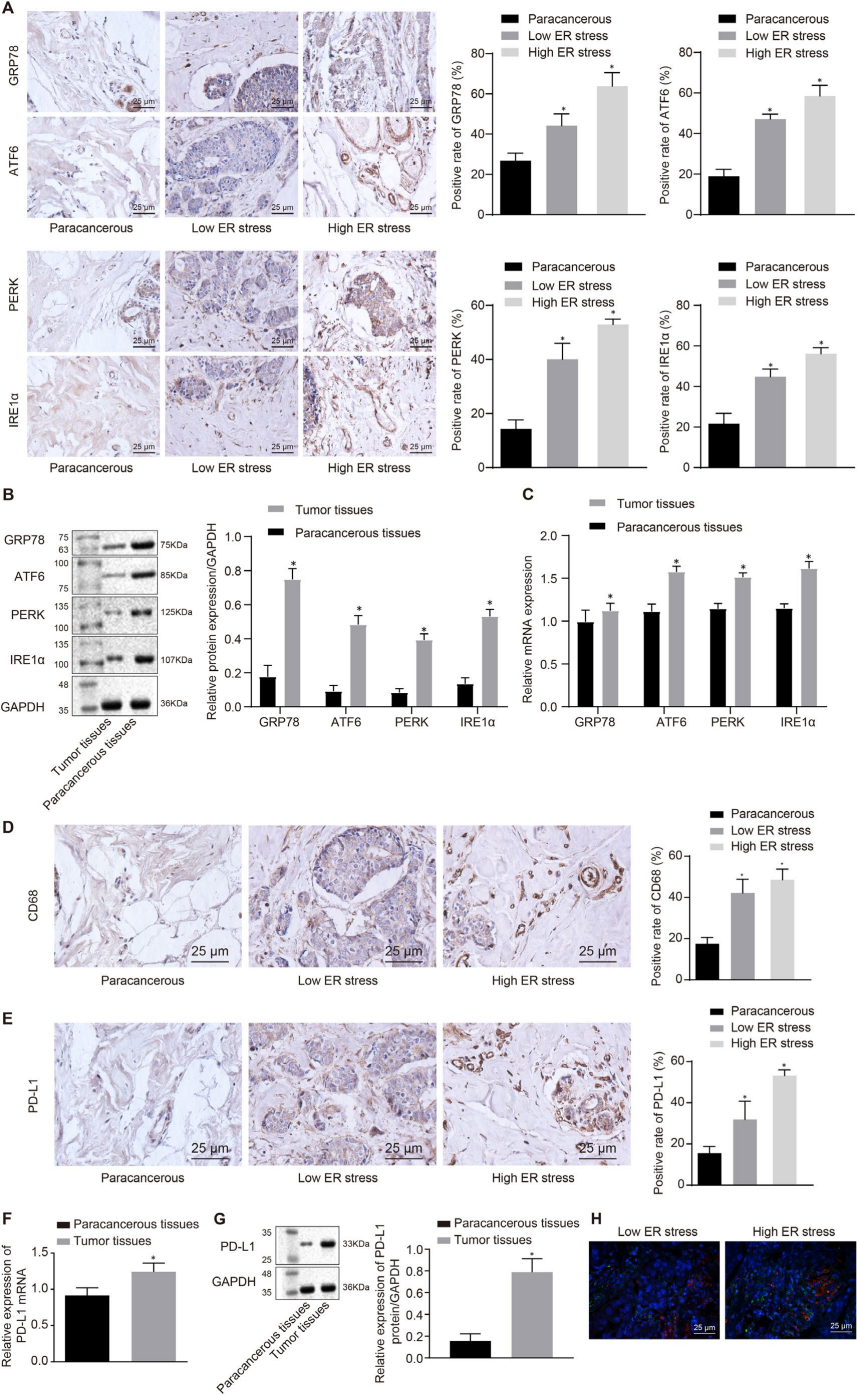
ER stress‐induced macrophage infiltration and contributed to PD‐L1 overexpression. A, GRP78, PERK, ATF6 and IRE1α protein expression in breast cancer tissues in response to low/high ER stress was determined using immunohistochemistry (×400). B, GRP78, PERK, ATF6 and IRE1α protein expression in tumour tissues and paracancerous tissues was determined using Western blot assay. *P < 0.05 vs paracancerous tissues. C, GRP78, PERK, ATF6 and IRE1α mRNA expression in tumour tissues and paracancerous tissues was determined using RT‐qPCR. *P < 0.05 vs paracancerous tissues. D, CD68 expression in response to low/high ER stress was assessed using immunohistochemistry (×400). E, PD‐L1 expression in response to low/high ER stress was measured using immunohistochemistry (×400). F, PD‐L1 mRNA expression in tumour tissues and paracancerous tissues was determined using RT‐qPCR. *P < 0.05 vs paracancerous tissues. G, PD‐L1 protein expression in tumour tissues and paracancerous tissues was determined using Western blot assay. *P < 0.05 vs paracancerous tissues. H, The co‐localization of PD‐L1, CD68 and macrophages in response to low/high ER stress was determined using immunofluorescence assay (×400). Measurement data were presented as mean ± standard deviation. n = 26. The unpaired *t* test was used to analyse the differences between two experimental groups

To evaluate the role of ER stress on breast cancer, firstly, the expression of ER stress‐related proteins: GRP78, PERK, ATF6 and IRE1α was measured in breast cancer tissues and paracancerous tissues. Immunohistochemistry results (Figure 1A) showed that tumour tissues displayed significantly elevated GRP78, PERK, ATF6 and IRE1α, relative to paracancerous tissues. Furthermore, an obvious increase in GRP78, PERK, ATF6 and IRE1α expression was noted in tumour tissues with high ER stress as compared to that with low ER stress. The same trend was observed in tumour tissues than that in paracancerous tissues determined using RT‐qPCR and Western blot assay (Figure 1B,C). These findings together indicated that ER stress was frequently activated in triple‐negative breast cancer.

Endoplasmic reticulum stress was previously found to exert antitumour effects via its role in immune regulation.^17^ We found ER stress‐related proteins to be interrelated with macrophage infiltration. Immunohistochemistry results showed markedly increased CD68 expression in tumour tissues relative to paracancerous tissues, and elevated CD68 expression was also observed in response to high ER stress as compared to low ER stress (Figure 1D). It has been reported that PD‐L1 mediates immune escape by inhibiting activation of T cells.^18^ We identified higher PD‐L1 expression in tumour tissues than in paracancerous tissues. PD‐L1 expression was also found appreciably increased in high ER stress when compared to low ER stress (Figure 1E). RT‐qPCR and Western blot assay (Figure 1F,G) similarly showed higher PD‐L1 expression in tumour tissues compared to paracancerous tissues. Immunofluorescence results demonstrated the co‐localization of PD‐L1 and CD68+ macrophage in tumour stroma of the high ER stress group (Figure 1H). Taken together, our findings showed ER stress‐induced macrophage infiltration and elevated PD‐L1 expression.

### 3.2 ER stress elevated breast cancer cell‐derived exosomal miR‐27a‐3p expression

**FIGURE 2:**
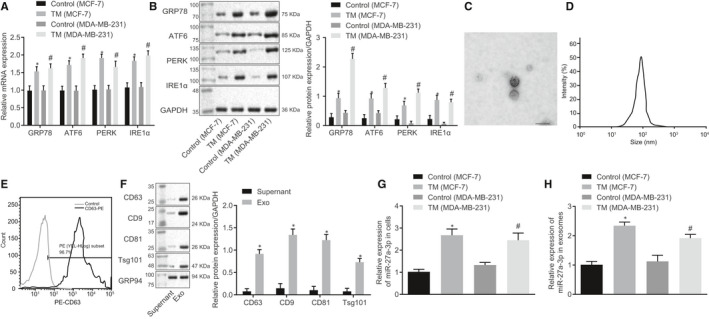
ER stress up‐regulated breast cancer cell‐derived exosomal miR‐27a‐3p expression. A, GRP78, PERK, ATF6 and IRE1α mRNA expression in breast cancer cells was determined using RT‐qPCR, *P < 0.05 vs normal cells. B, GRP78, PERK, ATF6 and IRE1α protein expression in breast cancer cells was determined using Western blot assay, *, vs normal tissues, *P* < 0.05. C, Transmission electron microscope was used to evaluate exosomes (scale bar, 100 nm). D, Diameter distribution detected using Image‐Pro Plus software. E, Flow cytometry was adopted to measure the content of exosome surface marker CD63. F, Contents of CD63, CD9, CD81, Tsg101 and GRP94 were observed using Western blot assay. G, miR‐27a‐3p expression was determined in MCF‐7 and MDA‐MB‐231 breast cancer cells, *, vs control (MCF‐7),* P* < 0.05; #, vs control (MDA‐MB‐231),* P* < 0.05. H, miR‐27a‐3p expression in MCF‐7 and MDA‐MB‐231 breast cancer cell‐derived exosomes was assessed using RT‐qPCR, *P < 0.05, vs control. I, miR‐27a‐3p expression in tumour tissues and paracancerous tissues. J, correlation analysis of miR‐27a‐3p expression and survival of breast cancer patients. Measurement data were presented as mean ± standard deviation. The unpaired *t* test was used to analyse the differences between two experimental groups, and ANOVA was utilized to analyse data among multiple groups, followed by Tukey's post hoc test

Exosomal miRs have been reported to be involved in macrophage infiltration and immune evasion.^19^ To investigate how ER stress modulates macrophage infiltration and PD‐L1 expression, we treated MCF‐7 and MDA‐MB‐231 breast cancer cells with tunicamycin to induce ER stress and then applied RT‐qPCR and Western blot assay (Figure 2A,B) to determine the expression of ER stressed related markers. As expected, GRP78, PERK, ATF6 and IRE1α expression was significantly increased in cells treated with tunicamycin as compared to the control. Exosomes were isolated from breast cancer cells and imaged using transmission electron microscope and dynamic light scattering detection (Figure 2C,D). Exosomes exhibited round‐ or oval‐shaped vesicles with 30‐120 nm diameter.

Flow cytometry (Figure 2E) was adopted to measure the level of exosome surface marker CD63, which was significantly increased. In addition, high expression of CD63, CD9, CD81 and Tsg101 were confirmed, along with poor expression of negative marker GRP94, in isolated exosomes using Western blot assay (Figure 2F, *P* < 0.05). These findings suggested exosomes were isolated successfully. miR‐27a‐3p expression in breast cancer cells MCF‐7 and MDA‐MB‐231 and in exosomes was determined using RT‐qPCR (Figure 2G,H, *P* < 0.05). Up‐regulated miR‐27a‐3p expression was found in both cancer cells and exosomes. miR‐27a‐3p up‐regulation was also noted in cells treated with tunicamycin as compared to the controls. Meanwhile, RT‐qPCR assay showed that miR‐27a‐3p was significantly up‐regulated in tumour tissues and was negatively correlated with patient survival (Figure 2I,J). Collectively, our results showed ER stress elevated breast cancer cell‐derived exosomal miR‐27a‐3p expression.

### 3.3 Breast cancer cell‐derived exosomal miR‐27a‐3p up‐regulated PD‐L1 expression in macrophages in vitro

**FIGURE 3:**
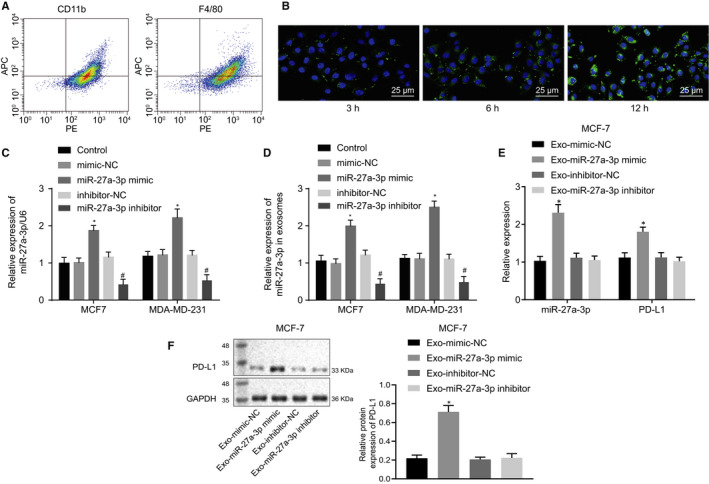
Breast cancer cell‐derived exosome‐encapsulated miR‐27a‐3p up‐regulated PD‐L1 expression in macrophages in vitro. A, Flow cytometry was used to validate PMA‐induced differentiated THP‐1 macrophages. B, Confocal fluorescence microscopy was used to observe the uptake of exosomes by macrophages (×400). C, miR‐27a‐3p expression in breast cancer cells was determined using RT‐qPCR, *P < 0.05 vs mimic NC; #, *P* < 0.05 vs inhibitor NC. D, miR‐27a‐3p expression in exosomes secreted by breast cancer cells was determined using RT‐qPCR, *P < 0.05 vs mimic NC. E, miR‐27a‐3p and PD‐L1 expression in co‐culture of exosomes and macrophages was determined using RT‐qPCR, *P < 0.05 vs Exo‐mimic NC. #*P* < 0.05 vs Exo‐inhibitor NC. F, PD‐L1 protein expression in co‐culture of exosomes and macrophages was assessed using Western blot assay. *P < 0.05 vs Exo‐mimic NC. #*P* < 0.05 vs Exo‐inhibitor NC. Measurement data were presented as mean ± standard deviation, and ANOVA was utilized to analyse data among multiple groups, followed by Tukey's post hoc test. Experiments were conducted in triplicates

To further elucidate the role of breast cancer cell‐derived exosomes in macrophages, we co‐cultured PKH‐67 (green)‐traced exosomes and PMA‐differentiated THP‐1 macrophages (Figure 3A,B). We found markedly increased red fluorescence signal within the macrophages at 12 hours after co‐culture, suggesting a substantial uptake of exosomes by macrophages.

After co‐culture for 12 hours, the uptake of PKH‐67‐labelled exosomes by macrophages was very obvious, indicating that exosomes can be transferred from breast cancer cells to macrophages. To further validate that breast cancer cell‐derived exosomes could deliver miR‐27a‐3p to macrophages, we overexpressed or knocked down miR‐27a‐3p in breast cancer cells MCF‐7 and MDA‐MB‐231. No significant differences were observed in exosomal miR‐27a‐3p expression between the mimic NC group and the inhibitor NC group. As expected, miR‐27a‐3p expression was markedly increased in breast cancer cells and exosomes in response to miR‐27a‐3p mimic as compared to mimic NC, while the opposite effect was noted in response to miR‐27a‐3p inhibitor as compared to inhibitor NC (Figure 3C,D).

To test whether transferred miR‐27a‐3p may regulate PD‐L1 expression in macrophages, we co‐cultured MCF‐7‐derived exosomes with macrophages. miR‐27a‐3p and PD‐L1 expression levels measured by RT‐qPCR were significantly increased in response to Exo‐miR‐27a‐3p mimic as compared to Exo‐mimic NC, but no obvious change was observed in response to Exo‐miR‐27a‐3p inhibitor or Exo‐inhibitor NC (Figure 3E, *P* < 0.05). Western blot assay (Figure 3F) revealed a marked rise in PD‐L1 expression in response to Exo‐miR‐27a‐3p mimic as compared to Exo‐mimic NC, while no significant change in PD‐L1 was observed in response to Exo‐miR‐27a‐3p inhibitor or Exo‐inhibitor NC. These data indicated that breast cancer cell‐derived exosomal miR‐27a‐3p up‐regulated PD‐L1 expression in macrophages in vitro.

### 3.4 Breast cancer cell‐derived exosomal miR‐27a‐3p up‐regulated PD‐L1 expression in macrophages in vivo

**FIGURE 4:**
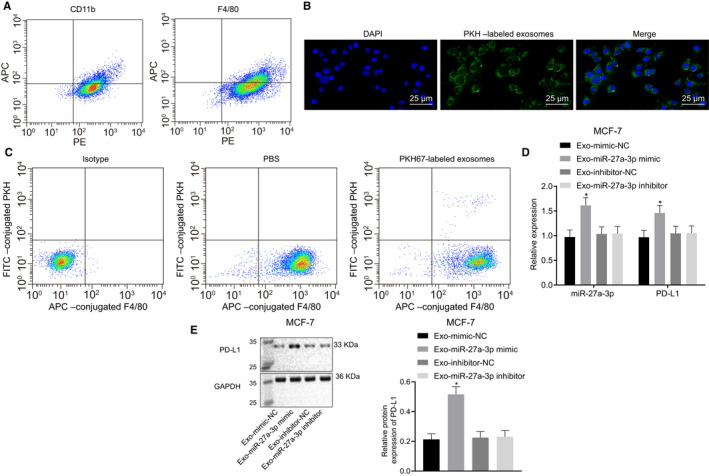
Breast cancer cells (MCF‐7)‐derived exosomes‐encapsulated miR‐27a‐3p elevated PD‐L1 expression in macrophages in vivo*.* A, Flow cytometry was used to evaluate macrophages. B, Confocal microscopy was used to observe exosomes uptake by macrophages (×400). C, Flow cytometry was used to determine exosomes uptake by macrophages; D, miR‐27a‐3p and PD‐L1 expression in co‐cultures of exosomes and macrophages determined using RT‐qPCR, *P < 0.05, vs Exo‐mimic NC. #*P* < 0.05 vs Exo‐inhibitor NC. E, PD‐L1 protein expression in macrophages co‐cultured with exosomes was determined using Western blot assay. *P < 0.05 vs Exo‐mimic NC. #*P* < 0.05 vs Exo‐inhibitor NC. Measurement data were presented as mean ± standard deviation, and ANOVA was utilized to compare data among multiple groups, followed by Tukey's post hoc test. Experiments were conducted in triplicates

To confirm the role of breast cancer cell‐derived exosomal miR‐27a‐3p in macrophages in vivo, we transfected different plasmids into breast cancer MCF‐7 cells and inoculated the transfected cells into nude mice. Next, we injected PKH‐67 (green)‐labelled exosomes into the nude mice every other day for 20 days. At 12 hours after the last injection, we separated peritoneal macrophages and examined them using flow cytometry (Figure 4A). Using confocal microscopy and flow cytometry, we found that PKH‐67‐labelled exosomes were enriched in the isolated peritoneal macrophages (Figure 4B,C), suggesting in vivo uptake of labelled exosomes by macrophages. RT‐qPCR results (Figure 4D) demonstrated that miR‐27a‐3p and PD‐L1 expression were significantly increased in response to Exo‐miR‐27a‐3p mimic as compared to Exo‐mimic NC, but no obvious change was observed in response to Exo‐miR‐27a‐3p inhibitor or Exo‐inhibitor NC. Western blot assay (Figure 4E, *P* < 0.05) revealed an obvious rise in PD‐L1 expression in response to Exo‐miR‐27a‐3p mimic as compared to Exo‐mimic NC, while no significant change in PD‐L1 was observed in response to Exo‐miR‐27a‐3p inhibitor or Exo‐inhibitor NC. These findings indicated that breast cancer cell‐derived exosomal miR‐27a‐3p up‐regulated PD‐L1 expression in macrophages in vivo.

### 3.5 miR‐27a‐3p targeted MAGI2 in macrophages

**FIGURE 5:**
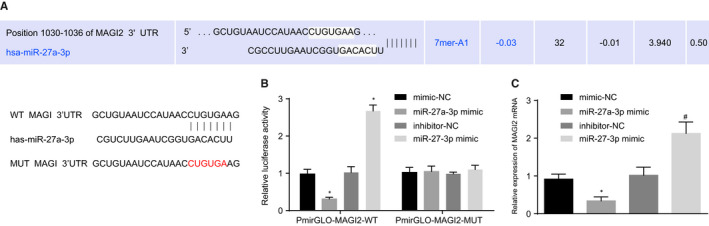
miR‐27a‐3p targeted MAGI2 in macrophages. A, TargetScan was used to predict the existence of putative binding sites between miR‐27a‐3p and MAGI2. B, Mutation of miR‐27a‐3p and MAGI2 binding sites and dual‐luciferase reporter assay of luciferase activity. C, MAGI2 expression in macrophages in response to miR‐27a‐3p mimic, mimic NC, miR‐27a‐3p inhibitor, and inhibitor NC was determined using RT‐qPCR, *P < 0.05 vs mimic NC. #*P* < 0.05 vs inhibitor NC. Measurement data were presented as mean ± standard deviation. Unpaired *t* test was used to analyse the differences between two experimental groups, while ANOVA was utilized to analyse differences among multiple groups, followed by Tukey's post hoc test. Experiments were conducted 3 times independently

To elucidate the regulatory role of miR‐27a‐3p, we predicted the existence of binding sites between miR‐27a‐3p and MAGI2 using TargetScan (Figure 5A). Dual‐luciferase reporter assay showed that co‐transfection of miR‐27a‐3p mimic and PmirGLO‐MAGI2‐WT decreased luciferase activity compared to mimic NC, whereas co‐transfection of miR‐27a‐3p inhibitor and PmirGLO‐MAGI2‐WT increased luciferase activity compared to inhibitor NC (*P* < 0.05), but no obvious change of luciferase activity was witnessed when co‐transfecting miR‐27a‐3p mimic or miR‐27a‐3p inhibitor with PmirGLO‐MAGI2‐MUT (Figure 5B). Moreover, using RT‐qPCR, markedly decreased MAGI2 expression was found in response to miR‐27a‐3p mimic treatment as compared to mimic NC, and MAGI2 up‐regulation was witnessed upon treatment with miR‐27a‐3p inhibitor as compared to inhibitor NC (*P* < 0.05, Figure 5C). Coherently, these data showed that miR‐27a‐3p targets and negatively regulates MAGI2 expression in macrophages.

### 3.6 MAGI2 inhibited PD‐L1 expression in macrophages by up‐regulating PTEN to inhibit PI3K/AKT signalling pathway

**FIGURE 6:**
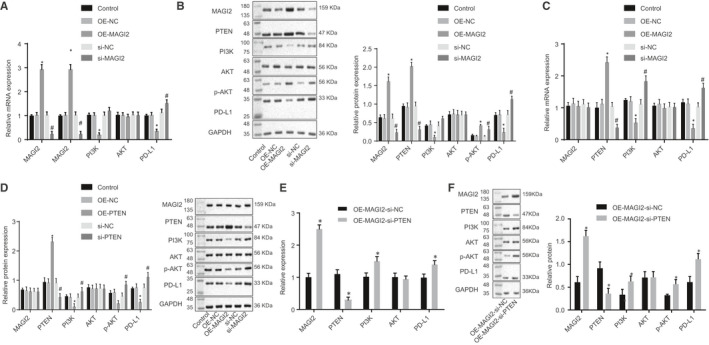
MAGI2 down‐regulated PD‐L1 expression in macrophages by elevating PTEN to inhibit the PI3K/AKT signalling pathway. A, MAGI2, PTEN, PI3K, AKT and PD‐L1 expression upon MAGI2 overexpression or repression in macrophages determined using RT‐qPCR, *P < 0.05 vs OE‐NC; #*P* < 0.05 vs si‐NC. B, MAGI2, PTEN, PI3K, AKT, p‐AKT and PD‐L1 expression was determined using Western blot assay upon MAGI2 overexpression or repression in macrophages, *P < 0.05 vs OE‐NC; #*P* < 0.05 vs si‐NC. C, MAGI2, PTEN, PI3K, AKT and PD‐L1 expression upon PTEN overexpression or repression in macrophages was determined using RT‐qPCR, *P < 0.05 vs OE‐NC; #*P* < 0.05 vs si‐NC, D, MAGI2, PTEN, PI3K, AKT, p‐AKT and PD‐L1 expression upon PTEN overexpression or repression in macrophages was determined using Western blot assay, *P < 0.05 vs OE‐NC; #*P* < 0.05 vs si‐NC. E, MAGI2, PTEN, PI3K, AKT and PD‐L1 expression after MAGI2 overexpression and PTEN repression in macrophages was determined using RT‐qPCR, *P < 0.05 vs OE‐MAGI2‐si‐NC. F, MAGI2, PTEN, PI3K, AKT, p‐AKT and PD‐L1 expression upon MAGI2 overexpression and PTEN repression in macrophages were determined using Western blot assay, *P < 0.05 vs OE‐MAGI2‐si‐NC. Measurement data were presented as mean ± standard deviation. Unpaired *t* test was adopted to analyse the differences between two experimental groups, while ANOVA was utilized to analyse differences among multiple groups, followed by Tukey's post hoc test. Experiments were conducted 3 times independently

It has been previously shown that MAGI2 regulates PTEN expression, while PTEN inhibits PI3K/AKT signalling pathway.^10^, ^13^ Thus, we hypothesized that MAGI2 may regulate PD‐L1 via the PTEN‐PI3K/AKT signalling pathway. To test this, we overexpressed or repressed MAGI2 expression in macrophages. We didn't find any significant differences in the expression of MAGI2, PTEN, PI3K, AKT and PD‐L1 between OE‐NC and si‐NC groups (*P* > 0.05). While MAGI2 and PTEN expression was increased, significant decreases were witnessed in PI3K and PD‐L1 expression (*P* < 0.05), and AKT expression exhibited no obvious change (*P* > 0.05) in response to OE‐MAGI2 compared to OE‐NC (Figure 6A). Western blot assay (Figure 6B) demonstrated that MAGI2, PTEN and phosphorylated (p)‐AKT expression was significantly increased, while PI3K, PD‐L1 expression was reduced (*P* < 0.05), and no obvious change was witnessed in AKT expression (*P* > 0.05).

Thereafter, we overexpressed or repressed the expression of PTEN in macrophages by transfection. RT‐qPCR results showed no significant differences in the expression of MAGI2, PTEN, PI3K, AKT and PD‐L1 between OE‐NC and si‐NC groups (*P* > 0.05). MAGI2 and AKT expression levels showed no obvious change in either OE‐PTEN or si‐PTEN groups when compared to OE‐NC or si‐NC, respectively (*P* > 0.05). Decreased expression of PI3K and PD‐L1 was observed in the OE‐PTEN group compared to OE‐NC, while increased PI3K and PD‐L1 expression was witnessed in response to si‐PTEN treatment compared to si‐NC (*P* < 0.05) (Figure 6C).

Using Western blot analysis, we did not find significant differences in MAGI2 or AKT expression between OE‐PTEN or OE‐NC groups, and between si‐PTEN and si‐NC groups (*P* > 0.05, Figure 6D). Similarly, decreased expression of PI3K, p‐AKT and PD‐L1 was observed in OE‐PTEN group as compared to OE‐NC, while increased PI3K, p‐AKT and PD‐L1 expression was witnessed in response to si‐PTEN treatment as compared to si‐NC (*P* < 0.05).

Furthermore, we overexpressed MAGI2 and simultaneously knocked down PTEN. RT‐qPCR results showed that AKT had no apparent change in response to OE‐MAGI2‐si‐PTEN treatment when compared with OE‐MAGI2‐si‐NC (*P* > 0.05, Figure 6E). There were significant increases in MAGI2, PI3K and PD‐L1 expression in response to OE‐MAGI2‐si‐PTEN treatment as compared to OE‐MAGI2‐si‐NC, whereas an opposite trend in PTEN, MAGI2, PI3K and PD‐L1 expression was witnessed in response to OE‐MAGI2‐si‐PTEN as compared to OE‐MAGI2‐si‐NC (*P* < 0.05). Western blot results showed that AKT showed no apparent changes in response to OE‐MAGI2‐si‐PTEN treatment vs OE‐MAGI2‐si‐NC (*P* > 0.05, Figure 6F). However, PI3K, p‐AKT and PD‐L1 expression were significantly increased in the OE‐MAGI2‐si‐PTEN group (*P* < 0.05). Taken together, MAGI2 inhibited PD‐L1 expression in macrophages via the PTEN/PI3K/AKT signalling pathway.

### 3.7 Macrophages activated by exosomal miR‐27a‐3p promoted the immune escape of breast cancer cells

**FIGURE 7:**
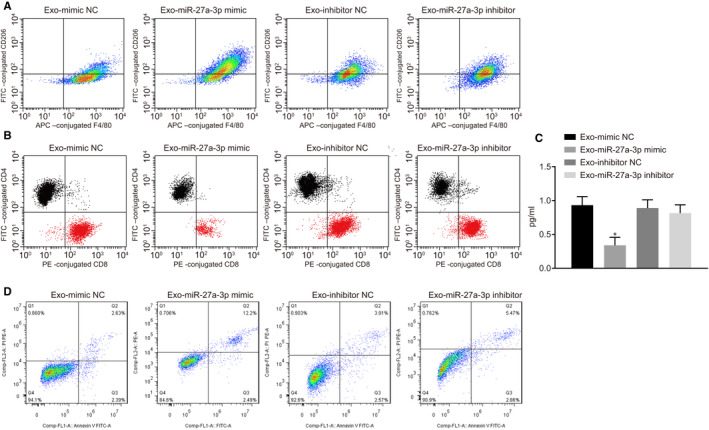
Immune evasion of breast cancer cells was enhanced by macrophages treated with exosomal miR‐27a‐3p. A, CD206 in macrophages was determined using flow cytometry. B, CD4 and CD8 in T cells were detected using flow cytometry. C, IL‐2 secreted by T cells was detected using ELISA assay. *P < 0.05 vs Exo‐mimic NC. D, Annexin V/PI assay was used to assess macrophages treated with exosomal miR‐27a‐3p on T cells apoptosis. Measurement data were presented as mean ± standard deviation. ANOVA was utilized to analyse differences among multiple groups, followed by Tukey's post hoc test. Experiments were conducted 3 times independently

Macrophages have been suggested to promote tumour progression by stimulating angiogenesis, enhancing tumour cell migration and invasion, and suppressing antitumour immunity.^20^ Therefore, we wondered whether breast cancer cells with ER stress could promote the M2‐polarization of macrophages, which may in turn impair cytotoxic CD8^+^ T cell responses. We isolated exosomes from MCF‐7 breast cancer cells and co‐cultured them with THP‐1‐differentiated macrophages. Flow cytometry revealed significantly increased CD206^+^ cells in response to Exo‐miR‐27a‐3p mimic treatment as compared to treatment with Exo‐mimic NC, but there was no apparent change in the number of CD206^+^ cells in response to Exo‐miR‐27a‐3p mimic vs Exo‐inhibitor NC (Figure 7A). These findings suggested that exosomal miR‐27a‐3p promoted macrophage M2‐polarization.

To describe the functional role of exosome‐treated macrophages, we co‐cultured macrophages with CD3^+^ T cells. We found a reduction of CD8^+^ T cells and decreased IL‐2 expression in T cells upon co‐culture with macrophages treated with Exo‐miR‐27a‐3p mimic as compared to Exo‐mimic NC. The proportion of CD8^+^ T cells and IL‐2 expression in T cells exhibited no obvious changes between macrophages treated with Exo‐miR‐27a‐3p inhibitor and Exo‐inhibitor NC (Figure 7B,C). Annexin V/PI assay showed more apoptotic T cells in macrophages treated with Exo‐miR‐27a‐3p mimic than in those treated with Exo‐mimic NC (Figure 7D). Taken together, we demonstrated that macrophage treatment with exosomal miR‐27a‐3p could lead to immune escape of breast cancer cells via inhibiting CD8^+^ T cells.

## 4 DISCUSSION

Chemotherapeutic resistance in certain subtypes of breast cancer is known as a cause of poor patient survival, and countering such resistance remains one of the major challenges in breast cancer management.^21^ At the same time, immune‐modulating treatments have shown efficacy for some breast cancers, given their immunogenic features.^22^ In this study, we identified a novel regulatory mechanism promoting immune escape of breast cancer cells. We found that under ER stress, breast cancer cells produce exosomes containing miR‐27a‐3p. Exosomal miR‐27a‐3p up‐regulates PD‐L1 in macrophages and promotes immune evasion of breast cancer cells by activating the PTEN‐AKT/PI3K pathway.

The first finding of this study was that ER stress was activated in breast cancer tissues, as evident by increased expression of GRP78, PERK, ATF6 and IRE1α in tumour tissues. In addition, ER stress‐induced macrophage infiltration and up‐regulated PD‐L1 expression. Endoplasmic reticulum stress was previously reported to be transmitted to myeloid cells, macrophages and dendritic cells, which are pivotal regulators of tumour immunity.^23^ Higher expression of GRP78, PERK, ATF6 and IRE1α^24^ has been noted in breast cancer tissues, indicative of stimulation of ER stress in breast cancer cells. It was previously reported that macrophages could stimulate angiogenesis, enhance tumour cell migration and invasion and thereby suppress antitumour immunity.^20^ Notably, the activation of macrophages is found to be enhanced by ER stress.^25^ PD‐L1 expression on tumour cells inhibits CD8^+^ T cell cytotoxicity and is shown as a prerequisite for immune evasion in immunogenic tumours.^26^ Marked elevation of PD‐L1 expression has been found to promote tumour growth and escape from antitumour immune mechanisms.^27^ In agreement with this finding, we found macrophage infiltration and PD‐L1 elevation in response to high ER stress in breast cancer cells, suggesting ER stress could induce immune evasion of breast cancer cells.

Exosomes are cell‐released, phospholipid‐enclosed vesicles, which act as biological cargos to carry and exchange biological materials or signals.^28^ Exosomes contain specific repertoires of miRNAs that can be functionally transferred to recipient cells.^28^ Notably, miR‐27a‐3p was up‐regulated in breast cancer cells and ER stress elevated miR‐27a‐3p expression shuttled by exosomes. Besides, breast cancer cells can deliver miR‐27a‐3p to macrophages through exosomes. Concordant with our study, miR‐27a was previously found up‐regulated in breast cancer cells and its mimics attenuated the anti‐proliferative function of metformin in the breast cancer cells by repressing tumour suppressor AMPKα2.^29^ Cancer cell‐derived exosomes have the capacity to modify the tumour microenvironment by influencing immune escape and metastasis, and moreover, exosomes‐loaded miRNAs are functionally implicated in cancer progression, metastasis and immune escape.^30^ For example, miR‐25‐93‐106b cluster plays a critical role in tumour metastasis and immune evasion via modulation of PD‐L1.^31^ In our study, we provided mechanistic evidence of exosomal miR‐27a‐3p in immune evasion in breast cancer through elevation of PD‐L1 and modulation of the PTEN‐dependent PI3K/AKT pathway.

We validated that MAGI2 is a target gene of miR‐27a‐3p. MAGI2 has been reported as an independent predictor of recurrence in prostate cancer.^32^ MAGI2, a scaffold protein required for PTEN, has also been identified as a target of miRNAs that are up‐regulated in tumours.^33^ The results of this study illustrated that MAGI2 up‐regulated PTEN to inactivate the PI3K/AKT signalling pathway. Consistently, PTEN enhancement has been found to underscore the MAGI‐2‐induced inhibition of cell migration and proliferation in hepatocarcinoma cells.^34^ A more recent study reported that MAGI2 binds to un‐phosphorylated PTEN and this interaction contributes to PTEN stability.^35^ Importantly, PTEN tumour suppressor is a major inhibitor of the PI3K/AKT pathway inactivation and a common target for suppressing multiple cancers.^36^ Inhibition of PI3K has been found to down‐regulate PD‐L1 expression and potentiate an anti‐proliferative effect, indicating that disruption of PI3K signalling may enhance the antitumour effect.^37^ We found increased phosphorylation of AKT and increased expression of PTEN in OE‐MAGI2‐treated macrophages, which down‐regulated PD‐L1. Thus, our results indicated MAGI2 inhibited PD‐L1 expression by up‐regulating PTEN and inactivating PI3K/AKT.

**FIGURE 8:**
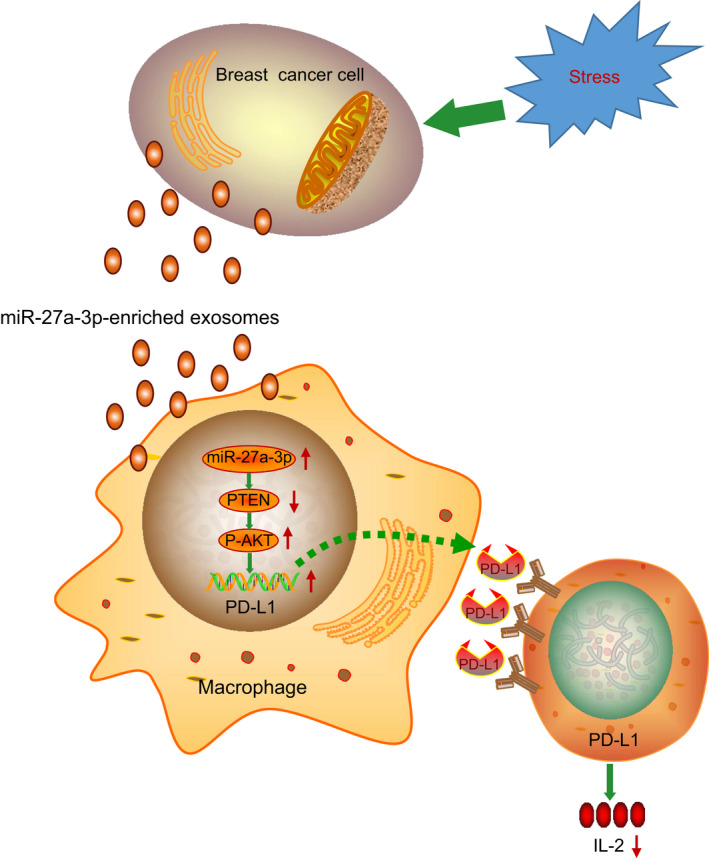
Mechanistic scheme depicting ER stress and immune escape via exosomal miRNA‐mediated cell‐cell communication. ER stress promotes exosomal miR‐27a‐3p from breast cancer cells and up‐regulates PD‐L1 expression in macrophages, thereby promoting immune evasion of breast cancer cells. ER stress‐induces the secretion of miR‐27a‐3p‐loaded exosomes from breast cancer cells. Exosomal miR‐27a‐3p up‐regulates PD‐L1 in macrophages and promotes immune evasion of breast cancer cells by activating the PTEN‐AKT/PI3K pathway

In conclusion, the present study provides new insights into the mechanism of miR‐27a‐3p on immune evasion of breast cancer cells. Notably, miR‐27a‐3p was up‐regulated in breast cancer cells and exosomal miR‐27a‐3p derived from breast cancer cells induced by ER stress up‐regulated PD‐L1 expression, thereby causing immune evasion via the PTEN/AKT/PI3K axis (Figure 8). The discovery of this mechanism may direct the future development novel therapeutic targets for breast cancer. However, further studies warranted to explore the role of this mechanistic pathway in clinical settings.

## CONFLICT OF INTEREST

The authors declare that they have no conflict of interest.

## AUTHOR CONTRIBUTIONS

Xiaoli Yao contributed to study idea, design and manuscript preparation. Yi Tu contributed to administrative support. Yulin Xu and Yueyue Guo contributed to data collection and interpretation. Xiaoli Yao and Feng Yao contributed to experiment performance and data analysis. Yulin Xu and Xinghua Zhang drafted manuscript. Xiaoli Yao, Yi Tu, Yueyue Guo and Feng Yao edited and revised manuscript. Xiaoli Yao, Yi Tu, Yulin Xu, Yueyue Guo, Feng Yao and Xinghua Zhang approved final version of manuscript.

## Data Availability

The data sets used and/or analysed in the current study are available from the corresponding author upon reasonable request.
